# Evidence of Cannabidiol Effectiveness Associated or Not with Tetrahydrocannabinol in Topical Administration: A Scope Review

**DOI:** 10.3390/ph17060748

**Published:** 2024-06-06

**Authors:** Ana Laura Masquetti Fava, Cinthia Madeira de Souza, Érica Mendes dos Santos, Luiza Aparecida Luna Silvério, Janaína Artem Ataide, Ana Cláudia Paiva-Santos, Jose Luiz Costa, Daniela Oliveira de Melo, Priscila Gava Mazzola

**Affiliations:** 1 Universidade Estadual de Campinas (UNICAMP), Faculdade de Ciências Médicas, Campinas 13083-887, Brazil; 2 Universidade Estadual de Campinas (UNICAMP), Faculdade de Ciências Farmacêuticas, Campinas 13083-871, Brazil; 3 Department of Pharmaceutical Technology, Faculty of Pharmacy of the University of Coimbra, University of Coimbra, Pólo das Ciências da Saúde, Azinhaga de Santa Comba, 3000-548 Coimbra, Portugal; 4 REQUIMTE/LAQV, Department of Pharmaceutical Technology, Faculty of Pharmacy of the University of Coimbra, University of Coimbra, Azinhaga de Santa Comba, 3000-548 Coimbra, Portugal; 5 Centro de Informação e Assistência Toxicológica de Campinas, Faculdade de Ciências Médicas, Universidade Estadual de Campinas (UNICAMP), Campinas 13083-970, Brazil; 6 Departamento de Ciências Farmacêuticas, Instituto de Ciências Ambientais, Químicas e Farmacêuticas, Universidade Federal de São Paulo (UNIFESP), Diadema 09972-270, Brazil

**Keywords:** cannabidiol, *Cannabis sativa*, topical administration, pre-clinical studies, clinical studies

## Abstract

*Cannabis sativa* is a plant of the Cannabaceae family, whose molecular composition is known for its vast pharmacological properties. Cannabinoids are the molecules responsible for *Cannabis sativa* potential effects, especially tetrahydrocannabinol and cannabidiol. Scientific development has shown interest in the potential of cannabidiol in various health conditions, as it has demonstrated lower adverse events and great pharmacological potential, especially when administered topically. The present study aims to carry out a scoping review, focusing on the use of cannabidiol, in vivo models, for topical administration. Thus, the methodological approach used by the Joanna Briggs Institute was applied, and the studies were selected based on previously established inclusion criteria. Even though more information regarding the dose to achieve pharmacological potential is still needed, cannabidiol demonstrated potential in treating and preventing different conditions, such as glaucoma, atopic dermatitis, epidermolysis bullosa, and pyoderma gangrenosum.

## 1. Introduction

*Cannabis* is a plant genus part of the Cannabaceae family, whose botanical morphology is known for the presence of hops, as much as the *Humulus* genus [1,2]. There are three family members of the *Cannabis* genus, such as *C. sativa* L., *C. ruderalis,* and *C. indica* Lam [2,3]. Although different classification is discussed among authors since the use is basically to distinguish the wild and domesticated variants. With that in mind, many authors refer to all *Cannabis* species as *C. sativa* only [2,4]. Furthermore, the leaves of the plants are easily recognized by their palmate form, with seven lobes and serrate leaflets, which differ according to genetic origin [5].

According to previous evidence, aside from the analgesia for pain control and anti-depressive (stress and anxiety relief), *Cannabis* and/or their bioactives demonstrate important pharmacological properties, such as anti-inflammatory, antitumor, antioxidant, antinociceptive, antimicrobial, neuroprotective, anti-convulsant, anti-parkinsonian, and reduction of blood pressure levels [6]. With those activities in mind, Cannabis presents the potential to be used in several diseases, but options are reduced in countries where Cannabis is illegal for adult and medical use.

Through the years, over 500 phytoactives were isolated and identified from *Cannabis*, including alkaloids, terpenoids, flavonoids, cannabinoids, and others [2,7,8,9,10,11,12]. Beside (−)-∆^9^-trans-tetrahydrocannabinol (∆^9^-THC) and cannabidiol (CBD), another 123 cannabinoids were identified, which are divided into classes according to their molecule similarity, including: (−)-∆^8^-trans-tetrahydrocannabinol (∆^8^-THC), cannabigerol (CBG), cannabinodiol (CBND), cannabielsoin (CBE), cannabicyclol (CBL), cannabichromene (CBC), cannabinol (CBN), cannabitriol (CBT), and miscellaneous types cannabinoids [7]. Those bioactives are produced by the glandular trichomes of the plant, whose pharmacological potential is contrasted in *Cannabis*, especially ∆^9^-THC, whose concentration is predominant [13].

CBD is known as the principal “non-psychotropic” compound of C. sativa and has been gaining more and more space in the scientific community due to its pharmacological potential use in neurological disorders [14]. CBD is proposed to be a negative allosteric modulator (NAM) on cannabinoid receptor-1 (CB1), which has a strong presence in the central nervous system. While CB1 receptors are primarily distributed in the central nervous system, CB2 receptors are more often associated with the immune system, however antagonistic effects on both receptors demonstrate a role in the stimulation of the inflammatory response. Many other non-CB1 and CB2 mechanisms have been proposed to explain the therapeutic effects of CBD, including its agonistic effect on serotonin 5-HT1A receptors and upregulation of anandamide signaling for psychiatric conditions. Further, the agonistic effect on vanilloid transient receptor potential vanilloid subtype 1 (TRPV1), μ and δ receptors for pain regulation, and inhibition of tumor necrosis factor-alpha (TNF-α) in inflammatory [15,16,17,18,19]. Due to these mechanisms of action, CBD can be used as an anti-aging, anti-inflammatory, antioxidant, and for pain relief for topical applications, for multiple sclerosis and neuropathic pain, and as an anticonvulsant for transdermal applications [15,16,17,20].

As CBD acts as a non-competitive inhibitor of the CB1 receptor, it antagonizes ∆^9^-THC’s psychoactive effect and subsequently improves the tolerability of concomitant THC use [15]. ∆^9^-THC is a partial agonist at post-synaptic sites of multiple proteins, including CB1, CB2, and glycine receptors, reducing excitatory transmission [19]. The distribution of the receptors with which ∆^9^-THC interacts leads to muscle relaxation, intraocular hypotensive agent, and pain relief, among others, when used as a topical agent [21,22]. When achieving systemic circulation, THC can also act as an antiemetic and antinociceptive [23,24].

**Figure 1:**
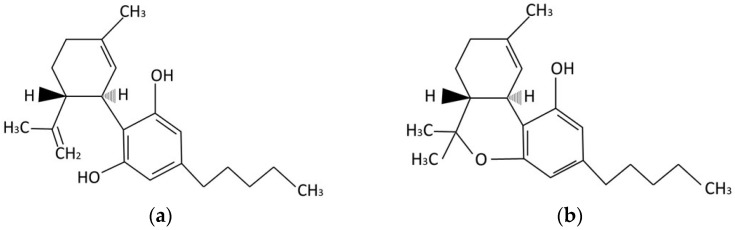
Molecule structure of (**a**) cannabidiol (CBD) and (**b**) (−)-∆^9^-tetrahydrocannabinol (∆^9^-THC). Figure was created by authors using ChemSketch Freeware 2021.2.0 (Advanced Chemistry Development, Inc., Toronto, ON, Canada).

∆^9^-THC, CBD, and its derivates are mostly apolar and considered hydrophobic/lipophobic molecules, as seen in Figure 1. Besides molecule characteristics such as polarity, administration pathway, and barrier constitution are some factors that influence active absorption. Topical administration involves applying a formulation directly to the skin or mucous membranes (i.e., ocular, nasal, buccal, rectal, pulmonary, and vaginal) to achieve a localized effect. However, if the active pharmaceutical ingredient (API) is absorbed and reaches the bloodstream, a systemic effect can be achieved, which is desired in the case of transdermal or transmucosal formulations [25]. Transdermal and transmucosal administration can be considered non-invasive or minimal invasive routes, which offer potential advantages over other routes, such as increased patient acceptance when compared with the parenteral route, higher bioavailability for some molecules and bypass of hepatic first-pass metabolism when compared with the oral route [26,27,28]. Topical administration, also, accumulates API in the site of action, reducing the risk of adverse events [29,30].

A simple search for “cannabidiol” at Clinicaltrials.gov returned 330 studies in February 2024, highlighting the efforts toward applying the medicinal potential of *Cannabis*. However, when searching for “topical cannabidiol”, only 11 studies returned, demonstrating that there are still doubts or missing information regarding this administration route.

The aim of this scoping review was to compile information in the literature regarding CBD pharmacological potential, associated or not with ∆^9^-THC, using topical administration as the main delivery path, and clinical or pre-clinical (in vivo) methodological approach. Furthermore, founded results were organized according to study type: clinical or pre-clinical, then topical or mucosal administration, and disease.

## 2. Results

### 2.1. Literature Selection

**Figure 2:**
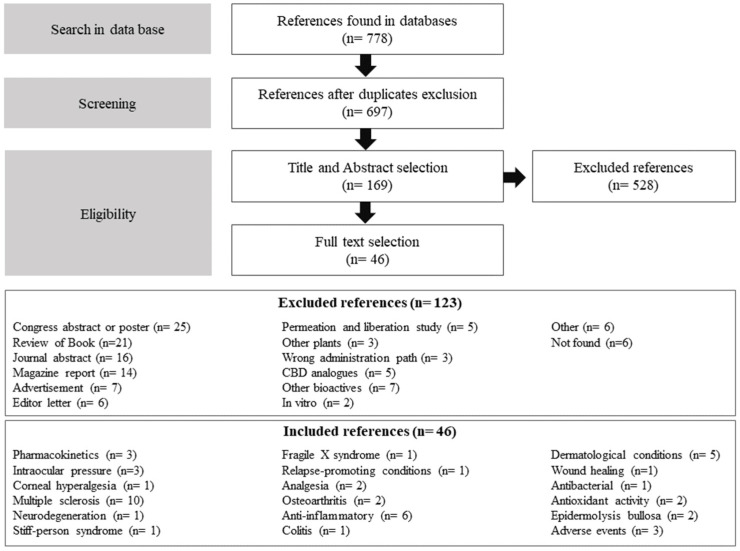
Methodological development and preliminary results.

The search in electronic databases resulted in 899 potential references, of which 107 were excluded for being duplicates (Figure 2). Then, at the first step, abstract inclusion, only 697 were analyzed by two authors, resulting in 183 inclusions. Therefore, the 183 references followed the second step, the full-text analysis, after which only 46 articles were included. The included references were published from 2004–2024 and classified by study type as pre-clinical when conducted with animals (*n* = 21, Table 1) and clinical when conducted with humans (*n* = 25, Table 2).

#### 2.1.1. Pharmacokinetics

Transdermal and transmucosal drug delivery depends on drug absorption into the systemic circulation through skin or mucous barriers, which may be affected by patients, drug, and formulation characteristics. This highlights the need for pharmacokinetic studies during formulation development that will evaluate the plasma levels of APIs after drug administration [74,75,76,77].

Hammell et al. [31] used a gel with 1% or 10% of CBD, administrated in different doses (0.62; 3.1; 6.2 and 62.3 mg/day) and observed the following plasma concentration of CBD: 3.8 µg/mL ± 1.4 µg/mL (in 9 rats, at 0.62 mg/day), 17.5 µg/mL ± 4.4 µg/mL (in 8 rats, at 3.1 mg/day), 33.3 µg/mL ± 9.7 µg/mL (in 8 rats, at 6.2 mg/day), and 1629.9 µg/mL ± 379.0 µg/mL (in 4 rats, at 62.3 mg/day), after 4 days of treatment.

In addition, Hannon et al. [32] found traces of CBD in plasma samples after six hours of transdermal formulation application. Concentration reached an amount of 12.8 µg/mL at the end of 1 week and 10.6 µg/mL at the end of 2 weeks. Results regarding THC detection demonstrated concentration values below the lower limit of quantification in both weeks [32]. The authors compared the pharmacokinetics of CBD and THC to its acid derivates, cannabidiolic acid (CBDA), and tetrahydrocannabinolic acid (THCA), respectively. CBDA showed mean concentrations of 32.4 µg/mL and 21.7 µg/mL in the first and second weeks, respectively, and THCA 3.8 µg/mL and 3.1 µg/mL, respectively. Statistical analysis confirmed that there was no difference in cannabinoid concentration when compared over the weeks. However, CBDA and THCA showed better absorption results than CBD and THC. Importantly, some animals experimented with erythema, a common side effect of transdermal ointment formulation, especially with extracts containing terpenes or ethanolic extracts that may cause skin sensitivity [32].

Considering previous studies, it is important to emphasize that differences in formulation content and administration path could affect cannabinoid pharmacokinetics. Therefore, Bartner et al. [33] compared 3 formulations of CDB: (i) CBD-infused transdermal cream, (ii) oral microencapsulated oil beads, and (iii) oral CBD-infused oil, and observed a dose-dependent behavior in maximal concentration (C_max_). Among the formulations, CBD-infused oil was the one with less inter-individual variability of CBD exposure and higher C_max_. In this study, lower C_max_ was observed for animals treated with transdermal cream, and significantly lower plasma concentrations were observed during evaluated time points.

Dose differences could influence the cannabinoid’s effects and its permeation into the skin, thus, the difference among studies could influence the comparison between results. Further, different animals and number of populations could influence data collection. Importantly, in the Gonzalez-Cuevas et al. [34] experiment, there was long-lasting CBD concentration in plasma, authors attributed this fact to its high lipophilicity, which allows it to stay longer available in the brain and plasma even for a few days past the treatment. Corroborating to the results of Hannon et al. [32], those who found traces of CBD, 6 h after treatment.

On another point of view comparing intranasal and transdermal administration paths, Paudel et al. [35] studied the CBD absorption and pharmacokinetics. First, nasal absorption of CBD was faster, identified in less than 10 min, and was detected after 0.5 min of treatment, specifically. On the other hand, transdermal absorption resulted in a mean plasma concentration of 6.3 ng/mL, which was attained at 15.5 h, and maintained for 48 h after gel application, declining only after 6 h of the gel removal. These results indicate a skin reservoir of CBD, due to its high lipophilic properties, that allows permeation through the *stratum corneum* but slow permeation through the dermis.

In this perspective, in treatments using CBD intranasal or transdermal formulation, if the need for CBD use is immediate, that is, the need to reach immediate results, intranasal is shown to be the best choice. On the opposite, in chronic treatments that require slow drug delivery, the transdermal pathway may be the best choice.

After a suspicious case reported by a driver, that was responsible for a car accident, claiming the use of *Cannabis sativa*-based medicine to treat his bruises, Hess et al. [51] conducted a study to verify the capacity of *Cannabis sativa* bioactives previously detected in the driver and absorption in the skin. In the driver, cannabinoids detected were 7.3 ng/mL of THC, 3.5 ng/mL of 11-Hydroxy-THC, and 44.6 ng/mL of 11-nor-9-carboxy-THC (THC-COOH), which confirms the presence of cannabinoids in blood circulation. During the experiment, volunteers using the same amount and the same formulation were tested for THC and its metabolites in blood samples, and nothing was detected. Possibly, the lack of identification was due to the concentration of the cannabinoids in the formulations administrated, since in analytical methods, the concentration of THC could be detected in both formulations tested 1.7 ng/mg of 11-hydroxy-THC and 102 ng/mg of 11-nor-9-carboxy-THC (THC-COOH) [51].

However, this study contraposes with the other studies previously discussed, in which skin absorption of *Cannabis sativa* bioactives has been detected, especially after authors determined improvement in the health conditions studied. Although the absorption may be dependent on the concentration of bioactive as much as formulation dependent.

#### 2.1.2. Intraocular Pressure

Predominantly, the studies aimed to determine the effect of cannabinoids on intraocular pressure (IOP), which is the pressure caused by the aqueous humor on the internal surface of the anterior eye. Pathologies associated with IOP included glaucoma, uveitis, and retinal detachment, especially if not well regulated [78]. The homeostatic mechanism to maintain the IOP is associated with the sympathetic nervous system, which influences aqueous humor secretion. The region where the regulation occurs is the juxta canicular region, when stressed the cells initiate a series of responses in a cascade leading to the increase of aqueous humor secretion [78,79].

∆^9^-THC applied topically reduced intraocular pressure in 30% after 8 h in male mice [36]. Although a different efficiency was observed in female mice, leading to a discussion that relied on gender dependency. This difference was confirmed by the level of mRNA expression in PCR, which showed a reduction of CB1 and GPR18 receptors in female mice. The intraocular pressure reduction by ∆^9^-THC use was given to the activation of CB1 and GPR18 receptors in combination. As for CBD, male and female wild-type mice showed elevation in IOP at 1 and 4 h, but in knockout CB1 mice, the CDB administration resulted in a reduction in IOP in the 1st hour and non-effect in the 4th hour. With those results, the authors discuss that CBD raises IOP CB1 dependently and did not affect IOP in animals with GPR18 antagonist O1918. On the contrary, the CBD increased the cannabinoid-related lipid species [36].

However, there are differences in IOP in mice and humans during the day and night. Humans have diurnal habits, thus, IOP peaking happens during the day, on the other hand, mice have nocturnal habits, with IOP peaking at night [38,80,81]. A study conducted by Rebibo et al. [38] considered these differences and evaluated mice IOP during the dark cycle. Blank nanoemulsion formulation did not affect IOP, while nanoemulsion with 0.4% CBD significantly decreased IOP in mice after 7 and 14 days of treatment, and with 1.6% CBD showed the same result after 14 days of treatment compared with the group baseline. On the contrary, the group treated with nanoemulsion containing 0.8% CBD did not show significant IOP reduction when compared with the initial baseline or blank nanoemulsion, leading to the conclusion of non-dose–response dependence for CBD activity in IOP reduction [38].

#### 2.1.3. Corneal Hyperalgesia

Neuropathic pain in the cornea may cause itch sensation, irritation, dryness, grittiness, burning, aching, and light sensitivity. Pain may be caused by corneal hyperalgesia which stimulates the nerve endings and induces hyper sensibility, usually, induced by moving air, minimal noxious stimulus, and normal light, which is also associated with allodynia [82,83,84,85].

According to the topical administration results, the application of 0.5% and 1% ∆^8^-THC reduced considerably the pain scores but did not prove to be effective in lower concentrations. CBD at 5% also showed a reduction in pain score but did not show effectiveness in pain reduction at 3%. Finally, 1.5% HU-308, a CBD derivative, proved antinociceptive effects [39].

When used separately CBD 5%, ∆^8^-THC 1%, and HU-308 1.5% demonstrated a reduction in neutrophil number, indicating a reduction of inflammation response. When the mechanism of action was evaluated using an antagonist AM251, it was found that ∆^8^-THC acts via the CB1 receptor to cause an antinociceptive effect, while on the other hand, even with antagonist administration, CBD 5%was able to reduce neutrophils in corneas. The CBD demonstrated an important action in 5-HT1A to reduce corneal pain and inflammation. As for HU-308, the mechanism of action was through the CB2 receptor [39].

With that in mind, the study presented strong information regarding the success of the use of ∆^8^-THC and CBD in corneal hyperalgesia to reduce pain and inflammation response [39]. Unfortunately, there were no other articles to compare their findings, although, the authors provided a novel subject to test cannabinoids as an option of treatment. Table 1 summarizes the methodological details of studies found about cannabinoids in IOP reduction and corneal hyperalgesia.

#### 2.1.4. Multiple Sclerosis (MS)

Multiple sclerosis (MS) is a disabling condition of the brain and spinal cords caused by an autoimmune response, in which the immune system does not recognize the myelin covering the nerves, responding with an attack mechanism, leading to nerve demyelination and inflammation process. Therefore, the flux of nervous impulses is compromised, reducing the responses after a stimulus and after a brain command. With time, the nerves deteriorate increasing motor and sensorial damage [86,87,88,89].

To study MS, scientists developed a model by inducing autoimmune encephalitis (EAE) in animals, by administration of soluble myelin-derived proteins, and myelin-derived peptides in adjuvant, or passive transfer of activated myelin-specific CD4 [90]. This model was used to evaluate the potential of CBD using a nasal delivery system (NDS) as a new potential treatment for multiple sclerosis [40].

In comparison, CBD demonstrated a decrease in encephalomyelitis score when associated with glatiramer acetate (CBD-GA) compared to medication for reduction of multiple sclerosis relapse, by the nasal delivery system, then in other administration paths [40]. Administration of CBD alone in the nasal delivery system reduced the expression of inflammatory cytokines, IL-6 and TNF-α, in the cerebellum tissue of encephalomyelitis mice. Similar results were obtained with the nasal delivery system combination of CBD-GA in the preventive use of prednisolone. Further, the nasal administration of glatiramer acetate (13.7 mg/mL) and prednisolone without CBD for preventive purposes reduced the inflammatory cell infiltration [40].

It is possible to assume that the combination of CBD-GA could bring benefits to MS treatment, including increasing neurogenesis, and could be considered for clinical trials as a novel treatment for multiple sclerosis condition [40].

The effectiveness of *Cannabis sativa* bioactives was observed in spasticity and neuropathic pain caused by MS:

- Spasticity

Bladder spasticity is an uncomfortable event caused by multiple sclerosis, among the symptoms is possible to cite urinary emergency and frequency, pain or discomfort, leakage of urine, and others. Administration of THC:CBD oromucosal spray showed an important reduction in the symptoms, according to the overactive bladder symptom score [52].

According to Maniscalco et al. [52]’s study, post-residual volume reduced after treatment started, as much as the MS spasticity, with a score improvement from 8 to 6 on the number rate scale. Moreover, 14 patients (out of 15) showed improvement of 20% or more at 0–10 number rate scale spasticity score measured after 4-week treatment, also mobility was improved from 33 to 24 s [52].

A treatment advantage was that no significant adverse events were reported by patients. These results showed that THC:CBD facilitated bladder emptying as the urodynamic showed a reduction of post-void residual volume and increased bladder volume and its compliance [52].

In accordance, Riva et al. [53] used the Modified Ashworth Scale, which rates the spasticity score, the results pointed to an improvement in patients treated with Nabiximol spray when compared to the placebo group. Nabiximol is another nomenclature adopted to refer to Sativex^®^, then, formulation and bioactives concentration remain the same [91].

Although a few patients discontinued treatment with Nabiximol due to adverse events, such as nausea and anxiety, disease progression, asthenia, dizziness, somnolence, vertigo, muscle spasticity or rigidity, and dry mouth. Even with these adverse events, in general, authors reported that in both phases Nabiximol was well tolerable [53].

- Neuropathic pain

Considering patients with an advanced MS condition, in the clinical trial conducted Rog et al. [54], the treatment with THC:CBD spray (2.7 mg: 2.5 mg) reduced neuropathic pain scale and numerical rating scale for pain. Intention to treat data analysis presented 89% of patients added reporting the dysesthetic pain and 11% painful spasms.

Other effects evaluated by the neuropathic pain scale pointed to improvement such as sleep disturbance. However, at least one adverse event was found in 30% of treatment patients, and only 68,8% in the placebo group, the most common event reported was nervous system disorders (dizziness) in treated and placebo groups. No fatalities, life-threatening incidents, or cases resulting in persistent disability or hospitalization were observed. Furthermore, there were no significant biochemical differences found in blood samples, nor were there any notable changes in vital signs. With that in mind, it is possible to assume that the treatment was well tolerable [54].

No significant differences in the neuropsychological outcomes were found by the Selective Reminding Test, although a unique difference was found in a mean improvement in the placebo group that did not match the treated groups. Then, the treatment with THC:CBD did not appear to significantly affect the MS-related neuropsychological outcomes measured. Furthermore, long-term use of THC:CBD did not show significant effects, it requires more studies to evaluate this information [54].

Clinical trials conducted by Nurmikko et al. [55] and Langford et al. [56] obtained similar results. Regarding Rog et al. [54] the study in long-term use of THC:CBD showed non-pain control and Nurmikko et al. [55] determined that long-term use (from the period of 871 days) of Sativex^®^ was effective in the maintenance of pain reduction. In addition, the group investigated Sativex^®^ effects in allodynia, but the differences between the control and treated groups were not significant. Furthermore, patients in the Sativex^®^ treatment group discontinued the treatment due to adverse events, non-compliance, and lack of efficacy [55].

Among the adverse events reported by patients, several gastrointestinal discomforts were noted, including nausea, vomiting, diarrhea, and constipation. Additionally, patients experienced nervous system-related adverse events, ranging from severe to mild-to-moderate psychiatric symptoms, as well as oral discomfort. Ischemic attack, a serious adverse event, was reported by one patient [55]. Gastrointestinal, oral discomforts, and nervous system affections (dizziness, impairment of balance, nausea, and intoxication feeling) were also reported as adverse events by patients in Rog et al. [57] and Langford et al. [56] clinical trials. Specifically, in the Langford et al. [56] study, phase A of the trial did not prove the effectiveness of THC:CBD spray in chronic neuropathic pain, although, the opposite was observed in phase B of the trial. This occurred since, in phase B, patients were asked to maintain a certain dose of the spray, a requirement not present in phase A, in that case, the analgesic effect was significantly higher.

On the other hand, a case reported by a 53-year-old patient with a relapsing multiple sclerosis diagnosis in 1999 was treated with the highest dose of tizanidine, baclofen, and benzodiazepines. In 2012, started a treatment with Sativex^®^, adjusting the optimal tolerance of 6 sprays a day. However, 4 weeks after treatment started patient presented convulsive seizures, thus, treatment with Sativex^®^ was discontinued. Discussing this case, the authors determined an impossibility of seizure induction by Sativex^®^ use, due to the patient’s conditions, there was a likelihood of such a situation happening caused by its previous conditions [58].

According to the literature information presented, neuropathic pain and spasticity were shown to be controlled using THC:CBD spray combination, including the commercial formulation. However, important reports of adverse events were mentioned and could lead to treatment discontinuation in long-term use. To minimize adverse events, an option is an adjustment in formulation, or even, the addition of co-medication to reduce the specific adverse events caused by the spray.

So, evidence pointed to a great response of CBD treatment in multiple sclerosis in both administration paths: mucosal and skin, especially for reduction in nerve preservation, inflammation reduction, and pain control.

#### 2.1.5. Neurodegeneration

The neurodegeneration process may be caused by a diversity of conditions, especially motor neuron diseases, such as Alzheimer’s, Parkinson’s, Multiple Sclerosis, Amyotrophic Lateral Sclerosis (ALS), and others, leading to progressive motor disability [92]. As seen before, previous authors studied the effect of CBD nasally applied in induced multiple sclerosis.

In the study conducted by Liput et al. [41], animals exhibited an intoxication level characterized by delayed righting reflex and ataxia. Results indicated a significant difference among groups treated with ethanol/vehicle and those treated with ethanol/1% CBD, suggesting a greater efficacy of the latter in mitigating ethanol-induced effects. However, despite these findings, statistical analysis revealed that treatment with CBD transdermal gel and transdermal vehicle did not produce alterations in either the intoxication effect or the pharmacokinetics of ethanol in both experiments.

Neurodegeneration in the entorhinal cortex detection assessed by Fluoro-Jade B (FJB), described as FJB+ cells, revealed statistically similar relevance in ethanol only and ethanol/vehicle gel groups, the same relevance was observed among animals treated with 1% or 2.5% CBD gels [41]. On the opposite side, 5% CBD gel treatment reduced by 48.8% the amount of FJB+ cells, which characterizes neuroprotective effects. In accordance with that, pharmacokinetics tests showed higher CBD plasma concentration at 5% than at 1% in the gel. Interestingly, diet showed interaction with CBD [41].

Further, FJB+ cells were found in neurodegenerative tissue along the entorhinal cortex, after four days with ethanol exposure. In fact, when compared to the control (ethanol only and ethanol/vehicle gel) the other groups with treatment collapsed since the control groups received the sample through different administration paths. Both CBD intraperitoneal injection and transdermal delivery showed a reduction of FJB+ cells when compared to its control groups, although, that was not statistically significant. Although it was possible to measure its neuroprotective effects, both showed similar neuroprotection capacity [41].

Information presented earlier proved that CBD associated with glatiramer acetate could be a great choice for multiple sclerosis treatment after intranasal administration in encephalomyelitis models [40]. In this case, after topical administration, the pharmacokinetics showed that the highest concentration of CBD in plasma was approximately 8.3 ng/mL [42].

During treatment, CBD-treated groups showed allergic reactions in the application area or the systemic level. However, the concentration in animals with encephalomyelitis treated with CBD showed great recovery throw time and less disability to encephalomyelitis when compared to untreated encephalomyelitis mice. In fact, encephalomyelitis mice treated with CBD cream presented a significant response in the needle test mechanical allodynia, when compared to the encephalomyelitis untreated group [42].

Histopathology assay of encephalomyelitis, untreated groups presented significant demyelination and axonal structures in the spinal cord, on the other hand, 1% CBD-cream treated groups showed reduction of the demyelination process and axonal loss caused by encephalomyelitis. In the same way, cell infiltration was detected in EAE untreated mice groups when compared to the encephalomyelitis treated with CBD groups, the last one, showed complete resolution of inflammatory cell infiltration [42]. These results indicate that CBD exhibits neuroprotective effects and can reduce neurodegeneration caused by inflammation.

#### 2.1.6. Stiff-Person Syndrome (SPS)

Stiff-person syndrome (SPS) is a rare autoimmune neurologic disease associated with high levels of autoantibodies against glutamic acid decarboxylase, a rate-limiting enzyme for the synthesis of gamma-aminobutyric acid (GABA). This syndrome is characterized by painful rigidity and muscle spasms [93,94].

A case report described by Vicente-Valor et al. [59] has Sativex^®^ (Sativex^®^, GW Pharma Ltd., Wiltshire, UK) introduced as a therapy to control extreme pain. Sativex^®^ is a commercial spray formulation of *Cannabis* extract, that contains, mostly, THC and CBD, but also, minor cannabinoids and other bioactives [91].

On stressful days, the patient self-adjusted his doses and achieved the maximum of 6 sprays without adverse events reported. Importantly, quality of life evaluation captures improvement; first, considering that before treatment patient was wheelchair dependent and suffered from muscle spasms and pain, in comparison, after treatment, the wheelchair was not needed, and the patient reported a reduction in pain. Then, there were improvements in gait and range of motion. Unfortunately, the SPS etiology remains uncertain, from the exams presented, the autoimmune-mediated mechanism is suggested by antibodies and other autoimmune comorbidities [59]. This case report may be a tool for further studies to consider Sativex^®^ as a treatment for pain control caused by this condition.

#### 2.1.7. Fragile X Syndrome (FXS)

A clinical trial was conducted by Heussler et al. [60] aimed to test a novel formulation of CBD gel in volunteers with Fragile X Syndrome (FXS), a rare genetic condition caused by the repetition of cytosine-guanine-guanine in the FMR1 gene on the X chromosome [60,95]. Safety and tolerability studies indicated that 85% of patients demonstrated at least one adverse effect such as gastroenteritis, vomiting, upper respiratory tract infection, or other. During the treatment period, 30% of patients experienced at least one adverse effect that could be related to CBD gel treatment. The adverse effects were resolved by dose adjustment. Other adverse effects classified as no serious included: gastroenteritis, vomiting, and upper respiratory tract infection in about 10% of patients.

However, the authors affirmed that ZYN002 (CBD gel) use was well-tolerable and clinically safe. Parameters such as echocardiograms, physical, neurological exams, and vital signs showed no relevant changes. Further, laboratory tests only reported an increase of eosinophil count after 83 days in patients with a moderate rash but normalized one month after the last dose administration [60].

In addition, subscales demonstrated great results after 12 weeks of treatment when compared to the screening. Anxiety, Depression, and Mood Scale subscales presented significant reductions in all parameters: manic/hyperactive behavior, social avoidance, general anxiety, and compulsive behavior. In accordance with these results, the depressive mood did not reach statistical significance. Further, the Aberrant Behavior Checklist-Community for FXS subscale, showed a reduction in its parameters, including stereotype, social unresponsive, irritability, hyperactivity, and inappropriate speech, as much as in the Pediatric Anxiety Rating Scale (PARS-S). On the other hand, a few parameters of the Pediatric Quality of Life Inventory (PedQLI) subscale did not reach improvement such as physical functioning, school functioning, and social functioning [60].

With that in mind, transdermal use of CBD in gel formulation manages to improve emotional and psychological symptoms related to Fragile X Syndrome patients, showing great bioactive bioavailability after absorption, and great tolerance after administration.

On the other hand, a study conducted by Scheffer et al. [61] aimed to evaluate the CBD transdermal gel treatment for children presenting epileptic encephalopathies. Overall, 46 patients were included in the study, and after 6.5 months of treatment, the median seizure reduction was 12.3%. Among seizure types, that showed relevant responses were focal impaired awareness seizures (44.5%) and tonic-clonic seizures (22.7%), compared to the baseline. In addition, 33 patients had both seizure types and showed a reduction of 43.5%, at 2 months, a reduction of 44.4%, and at 5 months, 57.7%. Concomitant use of clobazam improved seizure frequency. Furthermore, seizure severity, children’s behavior, and mood were also modified after the implementation of CBD transdermal gel therapy. Further, parents and/or caregivers rated qualitatively an important improvement in school, engagement or participation, cognition, energy, and other measurements [61].

Further, topical CBD for thumb basal joint arthritis-related pain resulted in significant improvements in pain, and disabilities of the arm, shoulder, and hand compared to the control. According to the score previously established, pain (rate of 0 to 10) was reduced from 5, at baseline, to 2 after CBD cream treatment, indicating 60% pain reduction (as lower the rate score, less disability). Also, disabilities of the arm, shoulder, and hand (rate of 0 to 100) were from 36, at baseline, to 22 with the CBD cream, referent to a 39% reduction [62]. Single assessment numeric evaluation (range 0 to 100) increased from 67.5 at baseline, to 78.5 with the CBD cream treatment, meaning a 16% increase in global well-being. Thus, this trial resulted in improvements in thumb basal joint arthritis-related pain and disability without adverse events [62].

#### 2.1.8. Relapse-Promoting Conditions

Other CBD effects were detected by Gonzalez-Cuevas et al. [34], in general, the cannabinoid exhibited positive effects in relapse-promoting conditions, such as stress, anxiety, and impaired impulse control. According to the results, CBD could be useful as a therapy for addiction treatment across several drugs of abuse, due to the reduction of vulnerability states that may cause relapse.

In alcohol and cocaine groups of rats, CBD was administered transdermally and showed a reduction of the yohimbine effect of stress induction which increases the drug seeking. When compared to vehicle groups, it demonstrated a reduction of reinstatement in cocaine and alcohol groups.

#### 2.1.9. Analgesia

In outpatients included in palliative care due to cancer, CBD administration has shown to be a great form of increased life quality due to pain reduction. Topical use of CBD was shown to be the most common application form, and most patients used CBD daily [63].

In accordance with these results, a clinical trial was conducted by Xu et al. [64], counting patients containing peripheral neuropathy secondary to diabetes mellitus, idiopathic peripheral neuropathy, and neuropathy secondary to medications (chemotherapy). Among those, one participant had a nonpalpable pulse and two had capillary refill time greater than three seconds. Of 29 patients, 23 presented no lower extremity edema, five showed light edema and one had severe edema.

The Neuropathic Pain Scale (NPS) score obtained across the weeks in both control and treated groups was 3.93 with a medium baseline score of 3.76, with reported sensations of surface pain, deep pain, and unpleasant pain [64].

Although no improvement was detected for deep pain, there was a significant reduction of NPS domains in the CBD group, such as intense, sharp, itchy sensations, and unpleasant surface pain. This corroborates the thought of CBD capacity in pain control, and most importantly, patients did not report adverse events [64].

#### 2.1.10. Osteoarthritis

Spontaneous osteoarthritis is related to genetic and mechanical impact as influence factors [96]. In general, these factors, in association, lead to cartilage wear out, especially after a stronger impact on joints, increasing the contact between bones in articulation areas [97], causing several pains, and impacting into patient’s life quality, due to the impossibility of performing basic daily life activities [97,98]. According to Brioschi et al.’s [43] study, the questionnaire results determine a significant reduction in pain severity score in dogs for spontaneous osteoarthritis (OA) after CBD administration, when compared to the control group after one, two, and four weeks of treatment.

Given the fact Brioschi et al. [43] introduced cannabinoid therapy in animals to test its capabilities for pain control, leading to quality of life. Along with the results described earlier, there was an observed increase in the quality-of-life index at all weeks when compared to the baseline. Comparing CBD groups with the baseline results, there was a significant decrease in the pain severity score after two and four weeks, leading to the conclusion that CBD may have reduced the pain after a regulated treatment [43].

As for the pain interference score, the values were lower at all weeks when compared to the baseline. The control groups did not show statistically significant variables at all the scores, but in general, there was a decrease in the pain severity score, pain interference score, and improvement in the quality-of-life index. In fact, the administration of firocoxib and prednisone did not affect CBD and control groups in most cases [43].

Mechanism of action, on the other hand, was not investigated, although, it was hypostasized that CBD may influence chronic pain, which intensifies if a non-steroidal anti-inflammatory drug (NSAID) is added since the inhibition of COX-2 would prolong the cannabinoids’ action [43].

#### 2.1.11. Anti-Inflammatory

Usually, topical skin drug administration is used to treat local conditions, and in this case, there is no interest in absorption or a minimum absorption. Although, that path can be used as a transdermal route, according to the formulation used.

Considering the pharmacokinetics studies, Hammell et al. [31] determined the potential anti-inflammatory effects of cannabinoids in the concentration found. Initially, permeation and distribution were considered adequate to the anti-inflammatory effect expected [99].

Hammell et al. [31] determinate the anti-inflammatory effect of CBD gel in monoarthritic-induced rats, using complete Freud’s adjuvant (CFA). After induction, there were noticed significant swelling in the side ipsilateral. Although, after four days of 6.2 mg/day and 62.3 mg/day CBD treatment, the knee joint showed a reduction of circumference, supposing a reduction in the inflammatory process, the opposite was seen with lower dose administration of CBD [31].

In accordance with the results, the histological evaluation showed that the synovial membrane was thickened after 7 days of intra-articular complete Freud’s adjuvant injection and reduced after 4 days of CBD 6.2 mg/mL treatment, and no changes were observed with low dose CBD administration [31]. In addition, the spontaneous pain was higher on day 3 in all animals, but after 4 days of 6.2 mg/mL CBD administration, the pain score reduced significantly, especially when compared to the control group [31].

In association with the anti-inflammatory effect, the CBD in 62.3 mg/day demonstrated a great reduction of pro-inflammatory cytokines such as TNFα when compared to the non-treated monoarthritic groups. Another important effect of CBD was the recovery of heat hypersensitivity in the rats’ paws, caused by the monoarthritic induction; further, CBD treatment did not alter the activity levels or motor abilities [31].

In addition, Mciver et al. [44], reinforced the anti-inflammatory effect of CBD by CB2 receptor suppression. In this case, the authors aimed to determine the wound healing potential of CBD extract, as pointed out, there were no differences in the daily mean wound area after treatments. The differences appeared after 7 days of treatment and the mean area decreased after 14 to 42 days. Overall, the healing process was slow until day 21 and increased faster after this period. On the contrary, there were no significant differences in the overall healing rate between any of the treatment groups, the same thing happens to total days to healing. Experiments demonstrated that inflammation reduction was probable due to CBD activation of the CB2 receptor; although, it did not demonstrate influence in the healing process [44].

In accordance with those results, pigs with 25 cm^2^ wound promoted by a thermocouple burn device heated to 100 °C were treated with topical Noneuphoric Phytocannabinoid Elixr 14 (NEPE 14), a formulation containing a full complement of phytocannabinoids (<0.3% Δ^9^-THC or CBD) and other phytochemicals. After 4 days of treatment, inflammatory cytokines, TNFα, and IL-6 were importantly reduced. However, the authors did not attribute the wound healing to the treatment, since both control and treated groups presented no difference in the healing process [45].

Furthermore, a study presented a possible multidirectional anti-inflammatory effect of CBD after topical application in UVA/B exposure skins. Plasma samples demonstrated downregulation of relevant phospholipids (lyso-PE and lyso-PC), prostaglandin, and thromboxane, although, it also demonstrated upregulation of anti-inflammatory lipoxin (LPXA_4_) [50].

For instance, Tubaro et al. [46] studied cannabinoids’ potential to reduce edema induced previously in animals’ ears and determined that cannabinoids considered psychoactive, such as Δ^9^-THC, Δ^8^-THC and Δ^8^-THCV (tetrahydrocannabivarin), reduced significantly edema with a dose-dependent response, meaning better results in the maximum concentration (1 µmol/cm^2^).

On the other hand, the non-psychoactive cannabinoids, CBC, CBD, CBCV (cannabichromevarin), and CBDV (canabidivarina) demonstrated lower potency in the highest dose (1 µmol/cm^2^) dose, with 40% of edema reduction for CBC and CBCV, 36% for CBD, and 29% for CBDV. When compared to the psychoactive cannabinoids the response was lower (20–70%), although the edema reduction was significant [46].

The authors used indomethacin as a comparative medicine that is usually used for anti-inflammatory effects and obtained an anti-inflammatory response of 22%-86% of edema reduction, and also dose-dependent. In comparison, the indomethacin group demonstrated better edema reduction and more potency than both cannabinoid groups; although, both cannabinoid groups demonstrated relevant edema reduction [46].

With these results, authors brought up the fact that the transdermal administration of CBD was effective against the inflammation caused by edema induced by carrageenan [100] and arthritis induced by complete Freud’s adjuvant Hammell et al. [31], and also reduced inflammatory mediators.

#### 2.1.12. Colitis

Colitis, a chronic inflammatory bowel condition, is characterized by blood and mucous diarrhea, with a non-determined etiology [100]. Therefore, Schicho and Storr [47] were the only ones to determine cannabinoids, specifically CDB, effects on colitis inflammation, administrated through the rectal mucosal and oral path to compare both effectiveness. After colitis induction, the results indicated that mice weight loss was associated with the condition and not with CBD or vehicle treatment. Intraperitoneal CBD administrations improved the colitis score and decreased myeloperoxidase (MPO) activity, demonstrating a reduction of inflammation and oxidative process [47].

In addition, the histological assessment presented lower epithelial lining destruction areas, reduction in colon thickness, and infiltration of monocytes compared to its vehicle. On the contrary, CBD treatment by intragastric administration did not improve colitis inflammation [47].

Although an interesting result regarding the intrarectal CBD treatment was obtained, a slightly important improvement in colon inflammation was seen, which was confirmed by the reduction in myeloperoxidase activity and the histological assessment showing a reduction in the leukocyte infiltration. Further, the crypt architecture was partially preserved in comparison to its vehicle [47].

According to the results obtained, the rectal administration of CBD could be a great choice for colitis treatment, even though, the path is still behind the CBD intraperitoneal administration [47].

#### 2.1.13. Dermatological Conditions

Dermatological conditions represent a fast-growing health concern, impacting a considerable segment of the population and leading to emotional and psychological challenges for affected individuals. These conditions stem from a multitude of factors, encompassing bacterial [101], fungal [102], parasitic [103], and viral presence on the skin [104], alongside weakened immune responses, allergens, irritants, genetic predispositions, and exposure to infected skin. Early detection plays a pivotal role in enhancing patients’ quality of life [105].

In recent years, there has been an increasing number of studies on cannabis for dermatological conditions, and below, some conditions will be discussed such as erythema, improvement in the skin surface, increased rise in skin hydration level, eczema, skin burns, and atopic dermatitis.

According to Ali and Akhtar [65], the use of a cream base containing 3% *Cannabis sativa* seed extract decreased erythema after 48 h of application, in comparison with the skin without the extract cream or control cream. Importantly, all volunteers presented non-irritancies, ensuring their safety for human use. Further, sebum treatment with pure base and seed extract base formulations decreased in the period of the study, although, with a marked decay of sebum after 3% *Cannabis sativa* seed extract use. Authors attribute this fact to the constituent of the *Cannabis sativa* extracts, including fatty acids and phytosterols, which inhibit 5-alfa-reductase, responsible for converting testosterone into dihydrotestosterone, metabolites that stimulate the skin sebum secretion [65].

More recently, the authors determined if *Cannabis sativa* extract would improve skin surface due to the preview results. Then, the study revealed that energy values were increased after dermocosmetic application for all volunteers, when compared to the base application, especially after 3 months. Although the other parameters such as contrast and variance effects for dermocosmetic were insignificant for the base. In this case, the parameter determined, energy, contrast, and variance should inform the improvement in skin texture [66].

The lack of contrast and variance is characterized by the increasing rise in skin hydration level, which creates a homogeny image. To support that, the surface evaluation of the living skin (SELS) results demonstrated that the base formulation did not produce significant effects, although, there were significant effects demonstrated by the statistical analysis for the results of dermocosmetic on SELS. In summary, the results pointed to a potential use of *Cannabis sativa extract* in the improvement of skin surface, and even in skin aging [66].

On the other hand, Maghfour et al.’s [67] study pointed out the extract’s potential use in eczema administration. First, at baseline patients scores by the Patient-Oriented Eczema Measure (POEM) demonstrated the severity of eczema, in which, 9 volunteers scored from 18 to 28 indicating severe to very severe eczema, 10 patients had a score from 8 to 16 indicating moderate eczema, and one volunteer scored 3 to 7 indicating mild eczema. After treatment, the questionnaire pointed out that from 16 volunteers, 3 presented reductions of their eczema from severe to mild range. The same result was observed with 3 volunteers who reported moderate eczema at the baseline, and at the end, they reported resolution of their skin disease after using topical CBD for 2 weeks. Further, all the volunteers experienced an improvement in the POEM score, significantly in the itch sensation, scaliness, and dry skin. Furthermore, Quality of Life Hand Eczema Questionnaire (QOLHEQ) scores, resulted in a significant reduction in anxiety, sadness, depression, and in embarrassment about their conditions after the treatment. CBD efficacy was rated by the volunteers, demonstrating at least 60% improvement, and no volunteers reported adverse events [67].

According to the results, it is possible to discuss the significant positive results caused by CBD in the treatment of atopic dermatitis, due to the suppression of cytokines involved in the inflammatory process [67].

One more use for the *Cannabis sativa* plant reported in the literature was in skin burn relief. However, no scientific background supports the use of the plant in skin burns, since the use was reported by local inhabitants of the North-West Frontier province in Pakistan, probably using the plant as a traditional therapy. According to the interview, 2 out of the 328 interviewed reported the use of *Cannabis sativa* L. crushed fresh leaves in skin burns, together with Allium cepa fresh scales, applied directly [68].

Recently, a randomized double-blinded placebo-controlled interventional pilot study determined the efficacy of CBD and aspartame formulation, as a novel topical treatment for atopic dermatitis. After 2 weeks of treatment, the investigator’s static global assessment reduced significantly, obtaining a score of 1.28 in the treated group compared to 0.70 in the placebo group. In addition, the CBD-only group did not obtain as much improvement as the CBD as the aspartame group, and the investigator’s static global assessment score was not significant compared to the placebo group. Besides the limitation of this study, due to the small sample size, the results pointed to important results that aspartame may be an interesting combination to improve CBD treatment in atopic dermatitis [69].

Therefore, it is possible to conclude that CBD has been used for various dermatological conditions, but further studies are still necessary to deepen its effectiveness and safety.

#### 2.1.14. Wound Healing

Healing functional skin after a wound is challenging due to the complex processes involved, including coordinated cell signaling and biochemical events [106]. Human skin naturally undergoes a four-stage repair process following damage, but disruptions at any stage can lead to impaired healing, potentially resulting in chronic wounds and affecting patients’ well-being [107]. Effective wound management is crucial to prevent complications, utilizing appropriate topical medications and dressings [108]. However, current conventional therapies, such as synthetic antibiotics and standard dressings, often fall short in treating chronic wounds, highlighting the need for advancements in medical technology in this area [109].

Case series reported the use of commercial formulations in patients with Pyoderma Gangrenosum (PG), which is a neutrophilic dermatosis responsible for the development of painful wounds [110,111]. Patients were treated with Bedrolite^®^ and Argyle™ (THC: CBD) in patient-dependent concentration and posology. Bedrolite^®^ is a *Cannabis*-derived product, considered CBD-only (it contains less than 1% THC and 9% CBD), leading to a less psychoactive product (Bedrocan^®^ International, 2021). Argyle™ on the other hand, are gel capsules containing THC and CBD in similar concentrations (Tweed Inc., Smiths Falls, ON, Canada).

Included patients did not show pain control with previously used corticosteroids. Cannabinoid medication use pointed to pain score reduction after a few weeks of treatment, although no wound healing improvement was reported [70]. The analgesic effect was attributed to the interaction of cannabinoids with receptors expressed on peripheral nociceptors and immune cells [70].

On the other hand, a case series study reported that CBD self-medication to painful wounds caused by Epidermolysis bullosa (EB) in children, patients reported blisters reduction, wound healing, pain, and itching sensation reduction [71]. Although the mechanism of wound healing was not elucidated, on the bright side, no adverse events were reported [71]. The same results were observed in patients using *Cannabis*-based medicine, containing THC and CBD [72].

Wound healing was also reported by Maida et al. [73], after the use of *Cannabis*. Ulceration in the legs in both reported patients was absolutely closed in a range of 75.6 days, and 50% closed in a range of 35 days. Patients demonstrated a similar period of healing and pain control, which was achieved after about 18–19 days after treatment began. In addition, planimetric wound image analysis showed that granulation dominates the first treatment half, and then, the second half showed reepithelization covering the granulated tissue.

Patients did not record side effects, which were attributed to its non-invasive and non-systemic capacity. Also, the benefits caused by the treatment were related to synergism among the bioactives present in the formulation: cannabinoids, flavonoids, and terpenes. The authors discuss that cannabinoids, such as CBD and THC, cause many physiologic effects that may improve healing [73].

With that in mind, studies are improving to determine the capacity of *Cannabis sativa* extract and/or THC and CBD in wound healing, including its mechanism of action.

#### 2.1.15. Antibacterial

Antimicrobial resistance poses a significant threat to modern medicine, where the effective prevention and treatment of bacterial infections are crucial. With bacteria evolving to resist antibiotics, the United States, Centers for Disease Control and Prevention (CDC) emphasized in a 2019 report that we are already in an era where antibiotics are becoming less effective, challenging the foundation of medical practices [48].

Considering the wound healing process discussed before, it is important to emphasize that wound contamination may accelerate the healing process by increasing the inflammatory activation in the first hours. In that case, if CBD causes systemic effects, it is likely that it reduced the number of bacteria and inflammatory activity, which caused wound healing retardation. So, if CBD is applied to an open wound, there are big possibility of systemic effects [44].

In fact, Blaskovich et al. [48] demonstrated that CBD reduced Gram-negative bacteria, including *N. gonorrhoeae*, one of the most resistant bacteria, in inoculated mice, after topical application. Further, in Gram-positive bacteria, CBD showed a high spectrum. The authors specified that the CBD repeatedly application did not lead to resistance.

The antibacterial effect is a different activity related to CBD with promising results; however, it would be necessary more experimental information to deepen this knowledge.

#### 2.1.16. Antioxidant Activity

This function is mostly related to the oxidative process caused by the ultraviolet (UVA and UVB) rays with sun contact. In summary, the ultraviolet rays are partly absorbed through the skin, inducing the generation of reactive oxygen species (ROS) leading to an oxidation reaction in cells. In the long term, ROS may promote DNA damage, which is associated with genetic mutations and, possibly, melanoma formation [112,113]. Cannabis sativa was described as an antioxidant after extract application or seed extract cream [65]. The antioxidant effect may influence dermatological conditions improvement, such as irritation, due to ROS inhibition [112,113].

According to Atalay et al. [49], when comparing the control groups (with non-exposure to UVA/B rays) to UVA/B exposure groups, the first one demonstrated a better number of certain proteins than the number of proteins in the second group. In this case, results indicate protein synthesis stimulation and protein degradation, respectively.

Cannabidiol treatment group, after UVA and UVB sun exposure demonstrated decreased pro-oxidant activity, not only by reducing the NADPH-dependent diflavin oxidoreductase 1, which generates superoxide anion radical, but also, by increasing the antioxidant enzymes expression such as Cu,Zn-SOD [49]. Further, CBD also improves the expression of ubiquination-related proteins, which regulates the proteins in cells. In fact, CBD affects the apoptosis paths by reducing the BcL-2 and the caspases recruitment, causing the opposite of UVA exposure effects [49].

On the other hand, Łuczaj et al. [50] provided information regarding the CBD effect in the changes of phospholipid and ceramide metabolism in plasma, after UVA/B exposure. In general, metabolic disturbance was prevented when CBD was applied, after UV radiation. According to the results, the downregulation of phospholipid types lyso-PE and lyso-PC indicates an anti-inflammatory effect of CBD, and the upregulation of phosphatidylethanolamines (PEo), which reduced the ROS property, indicates antioxidant stimulation [50].

Even though the study informed promising UV protective effects from CBD application, more information regarding this use is necessary to consolidate the potential use.

#### 2.1.17. Epidermolysis Bullosa (EB)

In general, the epidermolysis bullosa (EB) is a rare genetic condition characterized by the fragility of affected tissues. In this condition, any trauma causes painful ulceration, erosions, and mucocutaneous blistering [114]. Literature raised targeted the *Cannabis sativa* bioactives capacity for wound healing, besides pain control.

A case series conducted by Schräder [72] presented the improvement in wound healing of patients with EB after sublingual administration of *Cannabis*-based medicine containing THC and CBD. All patients did not present satisfactory effects with the previousview analgesia prescribed. Patients using sublingual CBM oil reported pain relief, reduction of pruritus, and urges to scratch. Although, one patient had to supplement the therapy with oxycodone after 6 months of single use of CBM oil [72].

A third patient reported side effects with previously prescribed treatment, leading to the use of CBM, which decreased pain and side effects. Specifically, this patient had skin carcinoma in the advanced phase, then, the concentration of CBM oil had to be adjusted, reducing pain in a few days after the adjustment. Unfortunately, the carcinoma caused metastasis and led to his death [72].

Considering the effects of CBM in patients with EB, it is possible to assume that it may increase life quality, by reducing pain from the mechanism already known. The combination of CBD and THC may strengthen medication, inducing the cannabinoids 1 and 2 receptors and induce beta-endorphin production. Further, CBD may also reduce the undesired effects of THC, such as sedation and intoxication [72].

### 2.2. Adverse Events

During this review, adverse events were reported, and the main events are summarized in Table 3. In addition, Schmitz et al. [115] compiled information regarding the adverse events reported after PlusCBD™ use. The PlusCBD™ is a brand name given to full spectrum hemp extract of ingestible and topical products [115]. During the period of their search (2018–2019), an amount of 1429 adverse events were detected in 1151 cases. The most common effects were due to topical administration and oral administration. In fact, among oral administration adverse events, indigestion, as much as abdominal discomfort, overcame any other topical administration related to adverse events [115].

Among the topical adverse events, the most reported are hypersensitivity, dermatitis, and rash. Other effects included a burning sensation, pruritus, pain, urticaria, dermatitis contact, and blister. Also, non-dermatologic effects after topical administration were not significant but included headache, anxiety, and abdominal discomfort. Further, 99.8% of the records reported were non-serious, and only 2 events were serious. Thus, the authors could conclude that the product proved to be safe and efficient when topically applied [115].

The first serious case was in an 83-year-old man, who experienced rash and hallucinations, after 2 months of PlusCBD™, in a spray dose of 1.5 mg of CBD/day. The results suggested that the product may not be the cause of the events since CBD is known to be non-psychoactive. The second case involved a 64-year-old woman reporting shortness of breath and tongue swelling after four sublingual doses of PlusCBD™ oil peppermint liquid. In that case, the patient was treated for a hypersensitivity reaction [115].

Further, 60% out of 48 patients treating epileptic encephalopathies with CBD reported treatment-related adverse events. Among the adverse events 70% (32 patients) were considered mild and 26% (12 patients) moderate. 5% of patients reported application site pain and somnolence, however, somnolence was reported by patients using clobazam in concomitance with CBD treatment. Adverse events caused by CBD were mostly related to the gastrointestinal system but did not lead to interruption or changes in the CBD treatment. On the other hand, there were no clinically significant changes in vital signs or electrocardiograms [61].

### 2.3. Limitations

The different methodological approaches used by the studies reviewed could interfere with the analysis, due to different experimental conditions. Thus, most of the comparisons were made by qualitative analysis.

## 3. Materials and Methods

It was performed a scoping review from February to March of 2021 and updated in February 2024, following the Joanna Briggs Institute’s approach for scope reviews [117].

### 3.1. Search Parameters

Only primary and secondary studies were included in the present review, without language delimitations. The period of publication was limited from 2004 to 2024. The reason for the parameter was based on the development of scientific and historical knowledge about cannabidiol and its worldwide use.

### 3.2. Search Strategy and Databases

Search terms were defined using MeSH terms and DeCS terms, previously to the search strategy development (Table 4). The search strategy was used in the following electronic databases: PUBMED, PUBMED PMC, BVS/BIREME (MEDLINE and IBECS), SCOPUS, Web of Science, EMBASE, Cochrane Library, EBSCOHost (MEDLINE, MEDLINE complete, Academic Search Premier, Regional Buseniss News, SocINDEX, Business Source Premier, Newswire, and SPORTDiscus).

#### Inclusion and Exclusion Criteria

References and their abstracts were migrated to Rayyan web application [118], where the duplicities were excluded. The selection was made in pairs by two authors (A.L.M.F. and C.M.S.) with blinds on. Once the selection was finished by both authors, the blinds were off and a third author (J.A.A.) solved the conflicts, according to the inclusion criteria.

Secondly, to be included articles should present in the abstract the primordial keywords and terms such as “Cannabidiol”, “Cannabis”, “Medical Marijuana”, “Administration, topical”, “Administration, Cutaneous”, “Transdermal Patch” and could present cannabidiol with one or more bioactives isolated. Furthermore, articles should be experimental.

After abstract selection, the included references were transferred to Excel 2019 (Microsoft Office), for full-text search. Full-text selection was made by one author, considered included the articles fitting the following criteria:

- Study type: Experimental clinical studies or pre-clinical with animals;
- Administration path: topical administration (mucosal or skin), also, studies could contain more than one administration path if discussed the different results between them;
- Formulation: Cannabidiol with or without other bioactives; in dosed *Cannabis sativa* extract or in commercial formulations isolated;
- Methodology: should contain the treatment used, according to the health condition studied;
- Although, articles were excluded if:
- Presented formulation containing more than one medicinal plant and/or which *Cannabis sativa* and/or cannabidiol is not the focus of the study;
- Presented formulation with other bioactives but not cannabidiol;
- Presented synthetic or natural molecules different from those in focus, especially if present contrary activities from those in focus;
- Full text not available or, if requested, not provided.

### 3.3. Data Extraction

Information was extracted by one author, considering the important information regarding the use of *Cannabis sativa* extracts or its isolated bioactives, such as health conditions, treatment regimen, formulation, other methodological approaches that affect the discussion, statistical analysis, main outcomes, and results, discussion, and conclusion. Finally, to organize references citations, references were extracted to EndNote X8.

## 4. Conclusions

This scoping review aimed to compile information in the literature regarding cannabidiol’s pharmacological potential, associated or not with tetrahydrocannabinol, using topical administration as the main delivery path and clinical or pre-clinical (in vivo) methodological approach.

In conclusion, the results pointed to the potential use of cannabidiol in topical administration in all conditions studied such as intraocular pressure, corneal hyperalgesia, osteoarthritis, colitis inflammation, multiple sclerosis, topical inflammation, relapse-promoting conditions, and neurodegeneration. Antibacterial and antioxidant activities were proven as well. However, CBD alone showed lower activity, but safety results would provide a potential use in humans, differently alone brought important adverse events, especially in the mucosal topical pathway. Thus, a THC and CBD combination could be a great choice of treatment since the synergy among the bioactives improved pharmacological potential and did not present important adverse events. Although, the methodological differences in the studies reviewed in the present study should be considered before moving to clinical trials.

Finally, this review presented relevant information regarding the known application of CBD by topical administration, increasing perspectives for future studies. More importantly, brought out the fact that: even though CBD is a potential and relatively famous bioactive, there are only 46 viable studies conducting experiments for topical administration. Thus, formulation details are relevant to new bioactive CBD products, since it may impact the absorption and efficiency of treatment.

With that in mind, new approaches could be applied in the future to increase CBD activity and efficiency. For instance, using pharmacological technology, such as nanoencapsulation, could improve drug delivery as much as establish a standard dose that could be assessed for different health conditions.

## Figures and Tables

**Table 1:** Methodological details included pre-clinical studies conducted with animals.

Animals	Condition	Intervention	Reference
Male Sprague Dawley rats	Pharmacokinetics induced monoarthritis	1% and 10% CBD gel Transdermal delivery 2 weeks	[31]
Six poupose-bred female research beagles	Pharmacokinetics	CBB/CBDA/THC/THCA ointment Transdermal delivery 2 weeks	[32]
Male beagle dogs, healthy conditions and sexually intact	Pharmacokinetics relapse-promoting conditions	CBD Trandermal cream 6 weeks	[33]
Male Wister rats	Pharmacokinetics	CBD gel Transdermal delivery 7 days	[34]
Male and female hairless rats IAF	Pharmacokinetics	CBD solution Nasal and transdermal delivery 4 and 48 h	[35]
Male and Female mice C57BL/6J (C57) strain except CB1 -/-	Intraocular pressure	Tocrisolve (THC and CBD) Eye 8 h	[36]
Male mice C57BL/6J (C57) strain except CB1 -/-	Intraocular pressure	(−)-∆^8^-THC-DMH with controlled deactivation Eye 5 h	[37]
Female C57BL/6 mice	Intraocular pressure	Nanoemulsions (CBD) Eye 2 weeks	[38]
Male BALB/c and CB2R knockout mice	Corneal hyperalgesia	Soybean oil (∆^8^-THC, CBD or HU-308 at 0.2–5% *w*/*v*) Eye 6 and 12 h	[39]
Female mice (C57B1/6) with 6–7 weeks of age.	Induced to present autoimmune encephalomyelitis	CBD Nasal delivery system 28 days	[40]
Adult male Sprague Dawley rats	Alcohol induced neurodegeneration with Ethanol	CBD 1%, 2.5% and 5% Transdermal gel 4 days	[41]
Male C57BL/6 mice	Induced to encephalomyelitis	CBD 2.5% O/W Cream 28 days	[42]
Dogs, with different breed, age, body weight and gender	Osteoarthritis	CBD oil Oromucosal 12 weeks	[43]
Standard bred horses	Wounds formation or scars on forelimbs, contaminated with fresh feces	CBD extract Topical	[44]
Female and male pathogen-free Yorkshire Hybrid pigs	Wound burn	CBD/THC cream Topical 7 days	[45]
Male CD1 mice	Induce inflammation with Croton oil dissolved in acetone	CBD solution Topical 6 h	[46]
Male CD1 mices (5–9 weeks old)	Colitis induction in mice with sulfonic acid	CBD Intrarectal, intraperitoneal or intragastric 32 h	[47]
Female outbred CD1 mice (UQBR-AIBN)	Contaminated back skin with bacterial inoculum in the concentration of 5 × 10^7^ CFU	CBD gel Topical 32 h	[48]
Nude male rats (RH-FOXN1RN)	Antioxidant	CBD Cream Topical 4 weeks	[49]
Male nude rats (RH-FOXN1RNU)	Antioxidant	CBD Cream Topical 4 weeks	[50]

CBD: cannabidiol, CBDA: cannabidiolic acid, THC: tetrahydrocannabinol, THCA: tetrahydrocannabinolic acid.

**Table 2:** Methodological details of included clinical studies conducted.

Study	Condition	Intervention	Reference
Case-control (N = 3)	Pharmacokinetics	Hemp oil crem Topical 3 days	[51]
Prospective pilot Study (N = 5)	Multiple sclerosis	THC/CBD spray Oromucosal 4 weeks	[36]
Prospective pilot study (N = 15)	Multiple sclerosis	THC/CBD spray Oromucosal 4 weeks	[52]
Multicenter, double-blind, randomized, placebo-controlled, parallel-group, phase 2 trial (N = 59)	Amyotrophic lateral sclerosis or primary lateral sclerosis	Nabiximol (THC/CBD) Oromucosal 4 weeks	[53]
Randomized, double-blind, placebo-controlled, parallel-group (N = 66)	Central neuropathic pain syndrome due to multiple sclerosis	THC (2.7 mg)/CBD (2.5 mg) spray Oromucosal 5 weeks	[54]
Randomized, double-blind, placebo-controlled parallel groups study (N = 125)	Unilateral peripheral neuropathic pain and allodynia	Sativex Oromucosal 6 weeks	[55]
Double-blind, randomized, placebo-controlled, parallel-group study (N = 93)	Multiple sclerosis	THC/CBD spray Oromucosal 1 year	[56]
Randomized double-blind, placebo-controlled, parallel-group study (N = 66)	Central neuropathic pain syndromes associated to multiple sclerosis	THC (21.6 mg)/CBD (20 mg) Oromucosal 2 Years	[57]
Case-report (N = 1)	Multiple sclerosis	Sativex Oromucosal 4 weeks	[58]
Case-report (N = 1)	Stiff-person syndrome	Sativex Oromucosal 14 month	[59]
Open label (N = 20)	Fragile X syndrome	CBD gel Transdermal delivery 12 weeks	[60]
Open label (N = 48)	Epileptic encephalopathies diagnosis	CBD 2% gel Transdermal delivery 26 weeks	[61]
Single-center, randomized controlled trial (N = 10)	Symptomatic thumb basal joint arthritis	CBD shear butter Topical 1 week	[62]
Cross-sectional (N = 58)	Analgesia	CBD Topical 3 months	[63]
Single-center, double-blind, randomized and placebo-controlled trial (N = 29)	Symptomatic peripheral neuropathy	CBD oil cream Transdermal delivery 3 weeks	[64]
Case-control (N = 11)	Erythema	3% *Cannabis sativa* extract cream Topical 12 weeks	[65]
Case-control (N = 11)		3% *Cannabis sativa* extract cream Topical 12 weeks	[66]
Observational (N = 16)	Diagnosed eczema	CBD Topical 2 weeks	[67]
Cross-sectional (N = 328)	Skin burns	Crushed fresh leaves Topical	[68]
Randomized double-blinded placebo-controlled interventional pilot study (N = 66)	Atopic dermatitis	CBD cream Topical 2 weeks	[69]
Case series (N = 3)	Pyoderma Gangrenos	Bedrolite^®^ and Argyle™ Topical 3 days	[70]
Case series (N = 3)	Epidermolysis bullosa	CBD oil cream Topical 2 to 3 times a day	[71]
Case series (N = 3)	Epidermolysis bullosa	CBD/THC oil Topican and sublingual	[72]
Prospective open label clinical trial (N = 2)	Wounds involving mucous membranes caused by Non-Uremic Calciphylaxis (NUC)	Cannabis based medicine Topical Until wound closure	[73]

CBD: cannabidiol, THC: tetrahydrocannabinol.

**Table 3:** Adverse events reported by pre-clinical and clinical included articles.

Administration Pathway	Formulation	Adverse Event	Reference
Eye	∆^9^-THC	Neurotoxicity	[116]
Oromucosal	CBD:THC spray	Nausea and anxiety, disease progression, and asthenia, dizziness, somnolence, vertigo, muscle spasticity or rigidity, and dry mouth	[53]
Oromucosal	CBD:THC spray	Nervous system disorders (dizziness)	[54]
Oromucosal	Sativex^®^ spray	Gastrointestinal discomforts (nausea, vomit, diarrhea, and constipation), nervous system (severe and mild-moderate psychiatric), and oral discomfort. Ischemic attack, a serious adverse event	[55]
Oromucosal	CBD:THC spray		[56]
Oromucosal	CBD:THC spray		[57]
Transdermal	CBD:THC	Erythema	[32]
Transdermal	CBD	Gastrointestinal discomforts	[61]
Topical skin	CBD	Allergic reaction in local or systemic	[42]

CBD: cannabidiol, THC: tetrahydrocannabinol.

**Table 4:** Search terms used in each database.

Plataform	Subject Vocabulary	Descriptors and Terms Used in the Search Strategy					
		1	2	3	4	5	6
PUBMED	MeSH—Medical Subject Headings	Cannabidiol	Cannabis	“Medical Marijuana”	“Administration, Topical”	“Administration, Cutaneous”	“Transdermal Patch”
PUBMED PMC	MeSH—Medical Subject Headings	Cannabidiol	Cannabis	“Medical Marijuana”	“Administration, Topical”	“Administration, Cutaneous”	“Transdermal Patch”
BVS BIREME	DeCS	Cannabidiol	Cannabis	“Medical Marijuana”	“Administration, Topical”	“Administration, Cutaneous”	“Transdermal Patch”
		Cannabidiol	Cannabis	“Marihuana Medicinal”	“Administración Tópica”	“Administración Cutánea”	“Parche Transdérmico”
		Canabidiol	Cannabis	“Maconha Medicinal”	“Administração Tópica”	“Administração Cutânea”	“Adesivo Transdérmico”
Scopus		Cannabidiol	Cannabis	“Medical Marijuana”	“Administration, Topical”	“Administration, Cutaneous”	“Transdermal Patch”
WEB OF SCIENCE		Cannabidiol	Cannabis	“Medical Marijuana”	“Administration, Topical”	“Administration, Cutaneous”	“Transdermal Patch”
EMBASE	Emtree	Cannabidiol	Cannabis	medical marijuana use preferred term: medical cannabis	administration, topical use preferred term: topical drug administration	administration, cutaneous use preferred term: cutaneous drug administration	“Transdermal Patch”
Cochrane Library	MeSH—Medical Subject Headings	Cannabidiol	Cannabis	“Medical Marijuana”	“Administration, Topical”	“Administration, Cutaneous”	“Transdermal Patch”

## Data Availability

No new data were created or analyzed in this study. Data sharing is not applicable to this article.

## Abbreviations

∆^9^-THC	(−)-∆^9^-Trans-Tetrahydrocannabinol
ALS	Amyotrophic Lateral Sclerosis
AO	Spontaneous Osteoarthritis
API	Active Pharmaceutical Ingredient
CBCV	Cannabichromevarin
CBD	Cannabidiol
CBDA	Cannabidiolic Acid
CBD-GA	Glatiramer Acetate And CBD
CBDV	Canabidivarina
CDC	Centers for Disease Control and Prevention
CFA	Freud’s Adjuvant
Cmax	Maximal Concentration
EAE	Autoimmune Encephalitis
EB	Epidermolysis Bullosa
FJB	Fluoro-Jade B
FXS	Fragile X Syndrome
IL-6	Interleukine 6
IOP	Intraocular Pressure
LPXA4	Anti-Inflammatory Lipoxin
MPO	Myeloperoxidase
MS	Multiple Sclerosis
NDS	Nasal Delivery System
NEPE 14	Noneuphoric Phytocannabinoid Elixr 14
NSAID	Non-Steroidal Anti-Inflammatory Drug
PARS-S	Pediatric Anxiety Rating Scale
PedQLI	Pediatric Quality OF Life Inventory
POEM	Patient Oriented Eczema Measure
QOLHEQ	Quality OF Life Hand Eczema Questionnaire
ROS	Reactive Oxygen Species
SPS	Stiff-Person Syndrome
THCA	Tetrahydrocannabinolic Acid
THC-COOH	11-nor-9-carboxy-thc
TNFα	Tumor Necrosis Factor
UVA	Ultraviolet Rays A
UVB	Ultraviolet Rays B
